# Effect of Calcination Temperature and Chemical Composition of PAN-Derived Carbon Microfibers on N_2_, CO_2_, and CH_4_ Adsorption

**DOI:** 10.3390/ma14143914

**Published:** 2021-07-13

**Authors:** Reyna Ojeda-López, Guadalupe Ramos-Sánchez, Cinthia García-Mendoza, Diana C. S. Azevedo, Ariel Guzmán-Vargas, Carlos Felipe

**Affiliations:** 1 Laboratório de Pesquisa em Adsorção e Captura de CO_2_ (LPACO_2_), Departamento de Engenharia Química, Universidade Federal do Ceará (UFC), Fortaleza 60455-760, CE, Brazil; diana@gpsa.ufc.br; 2 Departamento de Química, Universidad Autónoma Metropolitana-Iztapalapa (UAM-I), 09340 Mexico City, Mexico; gramossa@conacyt.mx; 3 CONACYT, Universidad Autónoma Metropolitana-Iztapalapa (UAM-I), 09340 Mexico City, Mexico; 4 Laboratorio de Nanotecnología, Centro de Investigación de Ciencia y Tecnología Avanzada de Tabasco (CICTAT), División Académica de Ingeniería y Arquitectura, Universidad Juárez Autónoma de Tabasco (UJAT), 86690 Tabasco, Mexico; cinthia.garcia@ujat.mx; 5 Laboratorio de Investigación en Materiales Porosos, Catálisis Ambiental y Química Fina, ESIQIE, Instituto Politécnico Nacional (IPN), 07738 Mexico City, Mexico; aguzmanv@ipn.mx; 6 Departamento de Biociencias e Ingeniería, Centro Interdisciplinario de Investigaciones y Estudios sobre Medio Ambiente y Desarrollo (CIIEMAD), Instituto Politécnico Nacional (IPN), 07340 Mexico City, Mexico

**Keywords:** carbon microfibers (CMFs), electrospinning, equilibration time, CO_2_ and CH_4_ adsorbed, open hysteresis loop

## Abstract

This work investigates the interplay of carbonization temperature and the chemical composition of carbon microfibers (CMFs), and their impact on the equilibration time and adsorption of three molecules (N_2_, CO_2_, and CH_4_). PAN derived CMFs were synthesized by electrospinning and calcined at three distinct temperatures (600, 700 and 800 °C), which led to samples with different textural and chemical properties assessed by FTIR, TGA/DTA, XRD, Raman, TEM, XPS, and N_2_ adsorption. We examine why samples calcined at low/moderate temperatures (600 and 700 °C) show an open hysteresis loop in nitrogen adsorption/desorption isotherms at −196.15 °C. The equilibrium time in adsorption measurements is nearly the same for these samples, despite their distinct chemical compositions. Increasing the equilibrium time did not allow for the closure of the hysteresis loop, but by rising the analysis temperature this was achieved. By means of the isosteric enthalpy of adsorption measurements and ab initio calculations, adsorbent/adsorbate interactions for CO_2_, CH_4_ and N_2_ were found to be inversely proportional to the temperature of carbonization of the samples (CMF-600 > CMF-700 > CMF-800). The enhancement of adsorbent/adsorbate interaction at lower carbonization temperatures is directly related to the presence of nitrogen and oxygen functional groups on the surface of CMFs. Nonetheless, a higher concentration of heteroatoms also causes: (i) a reduction in the adsorption capacity of CO_2_ and CH_4_ and (ii) open hysteresis loops in N_2_ adsorption at cryogenic temperatures. Therefore, the calcination of PAN derived microfibers at temperatures above 800 °C is recommended, which results in materials with suitable micropore volume and a low content of surface heteroatoms, leading to high CO_2_ uptake while keeping acceptable selectivity with regards to CH_4_ and moderate adsorption enthalpies.

## 1. Introduction

The nitrogen adsorption/desorption technique is ordinarily employed for the textural characterization of diverse materials synthesized to be used as CO_2_ or CH_4_ adsorbents. Specific surface area is one of the parameters of interest. Most of these materials require heat treatment to remove undesired species from the precursors used for their synthesis. Concerning carbon materials, regardless of the synthesis route, temperatures above 1000 °C are used to obtain a higher carbon content. However, depending on the target application, it is essential to preserve specific chemical functionalities and, to achieve this, temperatures below 1000 °C have been used, sometimes as low as 500 °C. When temperatures between 500 °C and 800 °C are used, a peculiar behavior has been observed for the nitrogen isotherms: unclosed hysteresis loops [1,2,3,4]. There are some possible reasons why this occurs [5,6,7]: (i) a nonrigid structure of carbon microfibers (CMFs) that causes deformation by adsorption or pore filling, (ii) trapped nitrogen cannot be released because of its affinity for the heterogeneous surface of the CMFs, i.e., the adsorption potential of the pore wall traps the nitrogen molecules, (iii) the existence of ink-bottle pores [6], (iv) the equilibration time during the adsorption/desorption analysis, or (v) a chemical composition with possible residues of small amounts of the precursor polymer. This study will focus on the last two hypotheses.

An open hysteresis loop has already been observed in carbon materials using other molecules as adsorbents. For example, Terzyk et al. studied the interaction of different molecules (methane, methanol, ethanol, carbon dichloride, and carbon tetrachloride) in microporous activated carbons, observing that, in the case of methane, the isotherms presented an open hysteresis loop; they concluded that at low coverages, methane adsorption takes place in micropores that are not particularly susceptible to oxidation [8]. In addition, they observed that as the temperature of analysis rises, the fraction of irreversibly adsorbed methane molecules also increases, which led to the following conclusions: (i) such an effect is connected to structural changes in the adsorbent (swelling) or to the rupture of weaker chemical bonds in the adsorbent network, leading to irreversible deformation, and (ii) the extent of the “memory effect” may determine the reversibility (closed hysteresis loop) or irreversibility (open hysteresis loop) of the adsorption process at low pressures [8].

The occurrence of an open hysteresis loop is highly dependent on both adsorbates and adsorbents. Wu et al. studied the effect of temperature on H_2_ and CO_2_ adsorption hysteresis in an MOF with ultramicropores. For H_2_ adsorption, the gap in the hysteresis loop reduces as the temperature increases and the loop is completely closed at 107 and 117 K [9], contrary to what Terzyt et al. observed for CH_4_ in carbon materials [8]. For CO_2_ adsorption, a similar behavior was observed: the hysteresis loop became closed at a higher analysis temperature, in which case the authors demonstrate that it is associated with the structural changes (or phase transitions) of the MOF [9].

Sircar et al. evaluated the effect of equilibration time on the hysteresis loop in N_2_, Ar, and H_2_ isotherms for MOFs and they concluded that: “(i) the degree of hysteresis between adsorption and desorption as well as the inflection point in the S-shaped isotherm were heavily dependent on the allowed time at each data point and also on the cumulative gas exposure time in N_2_ and Ar isotherms, and (ii) the adsorption isotherms do not change significantly when the allowed time (t_a,max_) is increased from 100 to 180 min, suggesting that additional allowed time will not lead to additional adsorption or hysteresis for H_2_ isotherms” [10]. On the other hand, Stoeckli performed a detailed study about the equilibrium time in water isotherms in activated carbons: he disclosed the importance of attaining the true adsorption equilibrium of water, in particular on oxidized active carbons, showing isotherms that took 10 days to 9 weeks to be fully measured where adsorption was very slow, especially at low relative pressures [11]. This work will also evaluate the impact of equilibration time on the hysteresis loop in the N_2_ adsorption isotherms on carbon microfibers, although—due to the limitations of keeping liquid nitrogen for several days—it was not possible to carry out measurements for more than 4 days.

To analyze the equilibration time, we must understand some technical details of the adsorption instrument used for this purpose, Quantachrome Autosorb 1 (Boynton Beach, FL, USA). The adsorption equipment software requires the input of the following parameters: the mass of the sample to be analyzed (previously degassed in vacuum); the type of analysis gas; the number of desired experimental points in the range of 0 to 1 relative pressure both in the adsorption and desorption curves; the equilibration time; and the desired tolerance for the sensed pressure. The equilibration time and pressure variation tolerance determine whether a given experimental point can be accepted as an equilibrium state in the adsorption or desorption isotherm [12]. The adsorption equipment has the option of choosing tolerance values between 0 and 9, such that a value of 0 means the smallest difference (typically 0.001 bar) between the selected pressure and the pressure reached experimentally in the adsorption equipment. On the other hand, a tolerance of 9 leads to a faster analysis, with less precise relative pressure values (normally 0.01 bar difference between the selected value and that reached experimentally) [12].

Regarding the equilibration time, the measuring instrument allows selecting from 1 to 99 min; for micropore measurements, it is recommended to use a tolerance of 0 and an equilibration time above 4 min [12]. It should be mentioned that both criteria (equilibration time and tolerance) must be met for the measuring instrument to take the reading as a valid equilibrium point. For example, suppose an equilibrium time of 10 min and a tolerance of 0 is selected. In that case, the point may be collected when 10 min have passed, as long as the pressure variations meet the requested tolerance. On the other hand, if the required tolerance is not reached before the required equilibration time (10 min) has elapsed, the point is discarded. This article will explore four equilibration times: 10, 20, 30, and 40 min with a tolerance of 0 (±0.001 bar).

**Figure 1:**
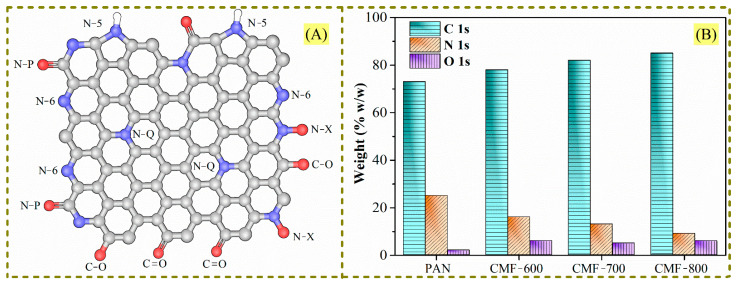
(**A**) Functional groups and (**B**) chemical composition (XPS results) in carbon microfibers.

In addition to evaluating the impact of equilibration time on the open hysteresis loop in carbon microfibers, we studied the effect of chemical composition. For this purpose, the isosteric enthalpy of adsorption was estimated using adsorption isotherms of CO_2_, CH_4_, and N_2_ at −10, 0, 10, 20, and 30 °C. In addition, a theoretical analysis by molecular dynamics (XC-GGA, density functional generalized gradient approximation) was used to determine the binding energy between CO_2_ or CH_4_ and carbon microfibers (considering graphene sheets with some functional groups of nitrogen and oxygen). In this work, some functional groups reported in the literature will be considered: pyridine-N (N-6), pyrrolic-N (N-5), pyridone (N-P), quaternary-N or graphitic-N (N-Q), pyridinic-N-oxide (N-X), C-C (sp^3^), C=C (sp^2^), C-O, and C=O (carbonyl group) [13,14,15]; they are shown in Figure 1A. The concentration of these species in each of the CMF samples was determined by X-ray photoelectron spectroscopy (XPS) analysis and reported in a previous work [13]. The broad spectra allowed the determination of the total content of carbon, nitrogen, and oxygen. Overall, the carbon content increases and nitrogen content decreases when the carbonization temperature is raised, while oxygen remains nearly constant (Figure 1B). Additionally, the high resolution spectra of N1s were obtained to define the different nitrogen functional groups, mainly the groups shown in Figure 1A [13].

## 2. Materials and Methods

### 2.1. Materials

Sigma-Aldrich (St. Louis, MO, USA) supplied polyacrylonitrile (PAN) polymer and anhydrous N, N-dimethylformamide (DMF, 99.8%), which were used as received without additional purification.

### 2.2. PANMFs and CMFs Synthesis

Polyacrylonitrile microfibers (PANMFs) were synthesized using polyacrylonitrile (PAN) and N, N-Dimethylmethanamide (DMF) with a concentration of 10%, as described elsewhere [16,17]. Briefly, the electrospinning apparatus was set at: (i) flow rate of 1.0 mL/h, (ii) voltage of 15 kV and (iii) distance between the tip of the syringe and the collector of 10 cm. The PANMFs obtained were carbonized at 600, 700 and 800 °C (all previously stabilized at 280 °C for 0.5 h in air atmosphere) for 1.5 h in nitrogen atmosphere [17]. The samples were labelled as: PAN (precursor polymer without any treatment), PANMFs (polyacrylonitrile microfibers), PANMF-E280 (polyacrylonitrile microfibers stabilized at 280 °C), CMF-600 (polyacrylonitrile microfibers stabilized at 280 °C and carbonized at 600 °C), CMF-700 (polyacrylonitrile microfibers stabilized at 280 °C and carbonized at 700 °C), and CMF-800 (polyacrylonitrile microfibers stabilized at 280 °C and carbonized at 800 °C).

### 2.3. Characterization Techniques

Fourier transform infrared spectroscopy (FTIR) experiments were performed on a Perkin Elmer Paragon 1000 (Waltham, MA, USA) in a 500 to 6000 cm^−1^ wavelength interval averaging 16 scans. The instrument used for thermogravimetry/differential thermal analysis (TG/DTA) was a Diamond TG/DTA Thermogravimetric Differential Thermal Analyzer (Perkin Elmer, Waltham, MA, USA) with a heating ramp of 10 °C/min in a range from 30 °C to 1000 °C. Powder X-ray diffraction (XRD) patterns were recorded on a Bruker D8 Advance (Billerica, MA, USA), using monochromatic CuKα radiation with a wavelength of 0.154 nm from 5.0° to 70.0° in the 2θ scale. Raman spectra of carbon fibers were obtained in a Horiba Jobin Yvon T64000 (Kisshoin, Minami-ku Kyoto, Japan) microspectrometer, using a 532.1 nm excitation wavelength, 20 mW power and 100× microscope objective region 50 and 1850 cm^−1^. SEM micrographs with magnifications from 1000× to 10,000× were obtained in a scanning electron microscope, JEOL JSM-6010LA (Akishima, Tokyo, Japan), at 20 kV acceleration voltage and high vacuum. TEM analysis was carried out in a high resolution transmission electron microscopy (HRTEM), Jeol 2100F (Peabody, MA, USA), at 200 kV acceleration. X-ray photoelectron spectroscopy (XPS) was performed with a Thermo Scientific K-alpha spectrometer (Waltham, MA, USA) using monochromatic radiation AlKα source (1487 eV). Instrument base pressure was 1 × 10^−^^9^ mbar.

Nitrogen adsorption/desorption isotherms were measured with a Quantachrome Autosob 1 instrument to assess textural properties. The samples were degassed at 200 °C for 12 h. The specific surface area was calculated by multiple-point Brunauer–Emmett–Teller (BET) [18,19], following Rouquerol recommendations for microporous materials [20,21]. Pore size distribution curves were computed using the nonlocal density functional theory (NLDFT) method for slit pore in carbon. CO_2_ and CH_4_ adsorption isotherms were also measured at −10, 0, 10, 20, and 30 °C. All samples were degassed at 200 °C, at a rate of 1 K/min under vacuum (5 × 10^−3^ mmHg) overnight, using the degassing system of Autosorb 1. The adsorption temperature was maintained (±0.1 °C) by circulating water/ethylene glycol from a constant temperature bath (MX07R-20 Model, PolyScience, IL, USA). Four equilibrium times were studied: 5, 10, 20 and 30 min. The equilibrium pressure tolerance was 0 (±0.001 bar).

### 2.4. Computation Details

Periodic DFT calculations were used to elucidate the role of graphene functional groups on the adsorption of CH_2_ and CO_2_ molecules. The Vienna Ab-Initio Software Package (VASP 5.4.4, 2017, Vienna, Austria) [22,23] was used for all calculations. Spin polarized calculations were performed using the projected augmented wave (PAW) pseudopotentials and the PBE exchange correlation functional [24]. The cutoff energy for the plane wave basis expansion was chosen to be 500 eV. A quasi-Newton algorithm was used to relax ions into their instantaneous ground state: the ions were allowed to relax but the cell size and shape was kept fixed. However, for different functional groups, the cell was allowed to relax shape and size without any adsorbent molecule. The adsorbate was allowed to interact with the substrate at a 3 Å distance from the surface close to the functional group. Note that the number of functional groups and combination of oxygen and nitrogen functional groups in carbon materials is vast; in this work, we aim to explore the effect of total oxygen and nitrogen functional groups, not to examine every possible functional group.

## 3. Results

**Figure 2:**
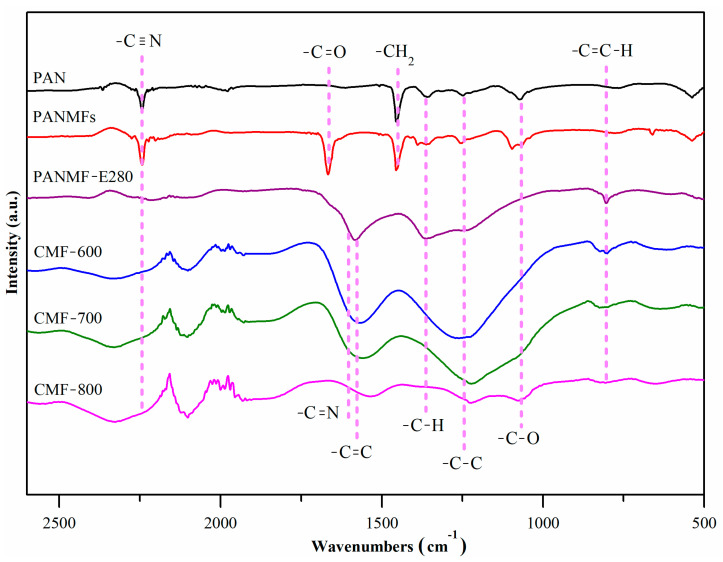
FTIR spectra of PAN, PANMFs and PANMFs stabilized and carbonized.

FTIR qualitatively evaluated the chemical composition of the samples, and Figure 2 shows the infrared spectra in the interval between 2500 cm^−1^ and 500 cm^−1^. The precursor polymer (polyacrylonitrile, PAN) had (i) two well defined signals: ~2245 cm^−1^ (nitrile groups, −C≡N) and ~1454 cm^−1^ (aliphatic groups, $\delta_{C-H}\;$ in CH_2_); and (ii) three weak signals: at 1360 cm^−1^ ($\delta_{C-H}\;$ in CH), at 1240 cm^−1^ (-C-C), and at 1070 cm^−1^ (-C-O) [25]. In addition to the five PAN signals, the PANMFs samples also presented one more signal in the region between 1600 cm^−1^ and 1700 cm^−1^, centered at ~1670 cm^−1^, ascribed to the carbonyl groups (-C=O). This could be due to residues of Dimethylformamide (DMF), which was used as a solvent to prepare the PAN microfibers, because the signal disappears when the sample was heated.

When PANMFs were stabilized at 280 °C, the intensity of the signal in ~2245 cm^−1^ (nitrile groups, −C≡N) decreased significantly and two broad and well defined signals were formed. The first one lay between 1480 cm^−1^ and 1630 cm^−1^, with a well defined center in 1574 cm^−1^ (-C=C); however, there were also the -C=N bonds within this broad signal, at 1590 cm^−1^. The onset of both bonds (-C=C and -C=N) suggests that cyclization and dehydrogenation had started; these processes co-occur during the stabilization of the materials at 280 °C (a temperature used in this work). The second signal was between 1180 cm^−1^ and 1420 cm^−1^, with two centers in 1240 cm^−1^ (-C-C) and 1360 cm^−1^ (-C-H). One weak signal could be observed at ~805 cm^−1^, which is characteristic of aromatic rings (C=C-H) and carbon-hydrogen bonds (-C-H, ~1360 cm^−1^) [26,27,28]. The decrease in the number of nitrile groups, loss of hydrogen and formation of aromatic structures are believed to result from the cyclization (-C=N-), dehydrogenation (-C=C-) and oxidation (-C=O) of PANMFs occurring during the stabilization stage.

The carbonization at 600 °C (CMF-600) and 700 °C (CMF-700) of the PANMFs led to: (i) increments in the signals between 1480–1630 cm^−1^ (-C=C, ~1574 cm^−1^ and -C=N, 1590 cm^−1^), although as the calcination temperature increased there was a trending shift towards the formation of -C=C bonds and a decrease in -C=N bonds, which is consistent with the XPS results reported in Ojeda et al. [13], showing that the amount of carbon increases as the amount of nitrogen decreases (Figure 1B); (ii) an increment in the signal between 1180–1420 cm^−1^, although the signal for -C-H (1360 cm^−1^) disappeared and the signal for -C-C bonds is shifted to a single center located at ~1240 cm^−1^. This corroborates that dehydrogenation had occurred, and adjacent polymer chains were joined by C-C bonds, resulting in longer carbon sheets, which is the objective of the 90 min carbonization process [17]. Nevertheless, when PANMFs were carbonized at 800 °C (CMF-800), both signals decreased because, at this temperature, the material had reached a higher carbon content and had significantly decreased in nitrogen compared to the CMF-600 and CMF-700 samples, which was confirmed by XRD and Raman analysis, as described in the following sections.

**Figure 3:**
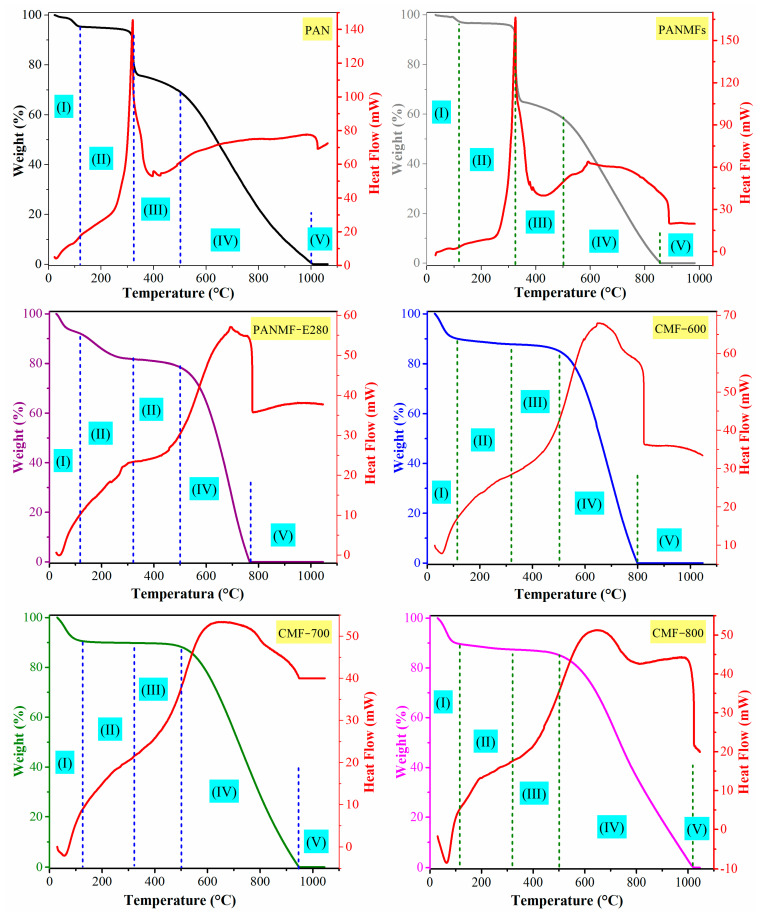
TG/DTA curves of PAN, PANMFs, and CMFs in nitrogen atmosphere: (I) desorption of physisorbed water, (II) cyclization and dehydrogenation, (III) dehydrogenation and denitrogenation, (IV) eliminating of some volatile components such as H_2_, CH_4_, CO, CO_2_, H_2_O, N_2_, NH_3_ and HCN, and (V) complete thermal degradation of PAN.

The TG/DTA curves for the studied samples are shown in Figure 3, and they were performed under nitrogen flow. All materials showed at least one exothermic and sharp peak in the DTA curve. In the case of PAN and PANMFs, this peak occured at 320 °C, above 320 °C, PANMFs showed a significant chemical transformation; therefore, it is recommended that the stabilization process for PANMFs be carried out at temperatures below 320 °C. The peak for the rest of the materials that had already been calcined appeared at temperatures above 500 °C (Figure 3), which accounts for the process of dehydrogenation and denitrogenation [29,30]. The increase in carbonization temperature generated broader peaks (CMF-800 > CMF-700 > CMF-600), which may be a result of a lower concentration of functional groups (as evidenced by XPS), so that the energy release became slower. A narrower peak means a large amount of heat evolved at a shorter time.

The TG curve in Figure 3 can be divided into five regions based on mass loss [25,31]. The first part is up to 120 °C, where the weight loss is mainly due to the desorption of physisorbed water. The second part is up to 320 °C, where PAN and PANMF materials experienced a more pronounced weight loss due to the occurring cyclization and dehydrogenation [29,30], PANMF-E280 experienced a smaller mass loss than PAN and PANMFs. On this very same range, the calcined materials (CMF-600, CMF-700, and CMF-800) presented minimum losses (less than 2% *w/w*), which means that the stabilization process had been successful on these materials. In the third part, up to 500 °C, in addition to dehydrogenation, denitrogenation took place. Materials that had not been carbonized (PAN, PANMFs and PANMF-E280) showed a larger mass loss in this temperature range (320 °C to 500 °C), while materials carbonized at 600, 700 and 800 °C showed a smaller percent loss. The fourth part accounts for temperatures above 500 °C and up to 800 or 1000 °C; the mass loss was due to eliminating of some volatile components such as H_2_, CH_4_, CO, CO_2_, H_2_O, N_2_, NH_3_ and HCN [29,30]. At this stage, all materials showed a mass loss of more than 50% *w/w*. Finally, beyond 800 °C (PANMFs, PANMF-E280, and CMF-800) or 1000 °C (PAN, CMF-700, and CMF-800) the thermal degradation of PAN had been completed. Considering the losses in the region between 120 °C and 320 °C, PANMFs lost 30% in mass, the characteristic region where the cyclization and dehydrogenation of the stabilization stage occurs; when the material was stabilized, the loss in PANMF-E280 decreased down to 10% and, for the case of the materials that were carbonized at 600, 700 and 800 °C, the loss was 2% in all cases, which is a sign of a good stabilization process. Likewise, the mass loss in the region between 320 °C and 500 °C was 3%, 2%, and 2%, respectively, for carbonized materials.

**Figure 4:**
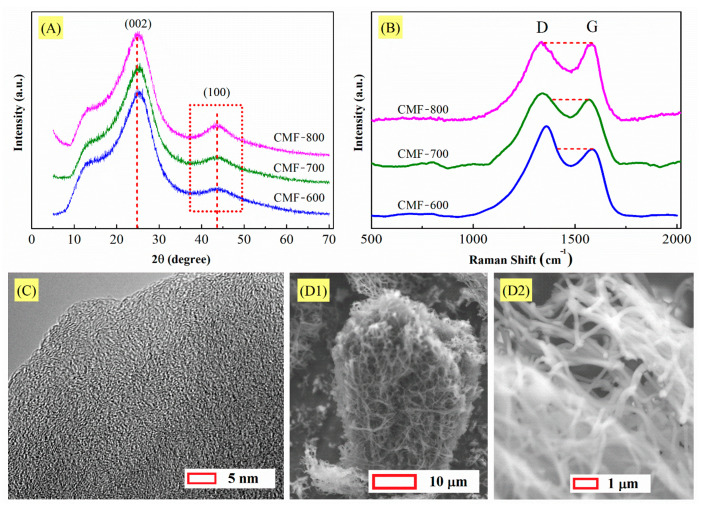
(**A**) XRD pattern and (**B**) Raman spectra for CMFs, “adapted from [13]”. (**C**) TEM and (**D1**), (**D2**) SEM micrographs of CMF-800.

XRD, Raman spectra, SEM images and TEM imagen for carbonized materials are shown in Figure 4. In the XRD patterns of Figure 4A, all materials exhibited the signal at 2θ = 25°, which corresponds to the graphitic (002) planes [13,32,33]. The high amplitude of the peak between 5° and 35° indicates that the structure of the carbon microfibers is composed mainly of amorphous carbon. This will be observed in the signal at 2θ = 44 ° in the plane (100), attributed to the lateral extent of graphitic domains. The spacing of the crystal layers ($d_{hkl}$) was calculated using Bragg’s Law [34]:

(1) $$n\lambda =2\;d_{hkl}\;sin\;\theta$$

where *λ* is the X-ray wavelength (Cu Kα: 0.154 nm), *θ* is the incident angle (the angle between the incident ray and the scattering plane), and *n* is an integer, in this case, *n* = 1.

From the XRD results, it is also possible to calculate the microcrystallite sizes (L) in the a-axes (stack width, La) and c-axes (stack highest, Lc) directions, as determined from the full width at half maximum (FWHM) of the diffraction peak according to the Scherer equation [35]:

(2) $$L=\frac{K*\lambda }{\beta *\cos\theta }$$

where *L* is microcrystallite size, *λ* is the wavelength of the incident X-rays (Cu Kα: 0.154 nm), *θ* is the incident angle (Bragg angle), β is the FWHM in radians, and finally, *K* is the Scherer constant, which depends on the lattice dimension, *K* = 1.84 for La ((100) plane) and *K* = 0.91 for Lc ((002) plane); the crystallite size along the a-axis, $L_{a\left(100\right)}$, corresponds to the extent of a hexagonal net plane, and the one along the c-axis, $L_{c\left(002\right)}$, to a width of stacking layer of hexagonal net layers [36,37]. Both parameters, the spacing of the crystal layers ($d_{hkl}$) and microcrystallite size (L), are present in Table 1.

The peaks of all materials vary only in the height of the signals, the centers being located almost at the same angle. Therefore, the value for d_002_ and d_100_ should be very similar in the three materials (Figure 4A). For example, d_002_ was 0.360 nm, 0.359 nm, and 0.358 nm for CMF-600, CMF-700, and CMF-800, respectively. Although there is minimal variation, d_002_ tended to decrease, which means a closer graphite interplanar spacing (0.3354 nm). Carbon materials with a turbostratic structure usually present a much larger spacing between adjacent layers than 0.3354 nm because of weak van der Waals interaction due to no regularity in stacking [37]. In general, heat treatment at high temperatures gradually decreased the value for d_002_, approaching 0.3354 nm, because the graphitic three dimensional stacking of layers occurs randomly in the crystallite; this parameter (d_002_) has commonly been used as a measure of the development of graphitic structure in carbon materials [37]. In the case of d_100_, the value was the same, 0.208 nm, for all materials. The decrease in FWHM when the carbonization temperature rose suggests an increase in their crystallinity; furthermore, $L_{c\left(002\right)}$ and $L_{a\left(100\right)}$ presented the same behavior and this increase in crystal size can be interpreted as a decrease in the disorder between the carbon sheets in the carbon microfibers.

Based on the XRD results, it can be observed that due to the temperature used for the synthesis of the materials, the degree of amorphicity is high. Raman spectroscopy results confirm this point. Generally, this technique allows to characterize the structure development of carbon material. The spectra in Figure 4B indicate a structural change in the CMFs with increasing carbonization temperature. Two peaks can be identified, G (Graphite) and D (Disorder). The G band centered at 1580 cm^−1^ is associated with the sp^2^-hybridized carbon, and the D band at 1360 cm^−1^ is attributed to the disorder related to the sp^3^-hybridized carbon. The intensity ratio of the D band to G band (I_D_/I_G_ = R) expresses the sp^3^/sp^2^ carbon ratio, indicating the degree of disorder. It depends on the degree of graphitization and the alignment of the graphitic planes. The R-value is sensitive to the ratio of the concentration of graphite edge planes and/or crystal boundaries relative to standard graphite planes, i.e., the lower the R-value, the higher is the amount of sp^2^ (graphite) clusters that exist on the sample; there is an empirical formula for the relationship between the R-value and the crystallite domain size, $L_{a}$, of graphite as $L_{a}=4.4/R$ [32,38]. Both R and La were calculated, and they are shown in Table 1. The R values are relatively high as compared to those usually presented by carbon materials with a higher degree of graphitization (lower than 1.0) [32,38], which means that the I_D_/I_G_ ratio is not conclusive at these low carbonization temperatures. Nevertheless, R-values corroborate the findings previously described by several studies about carbon: they decrease with an increase in carbonization temperature. By simple visual inspection, it can be observed that the G-band signal increases while the D-band signal decreases. In the case of carbon microfibers synthesized from PAN and by the electrospinning method, Andrei et al. have shown that temperatures above 1200 °C allow obtaining materials with a significant degree of graphitization and values close to 1.0 for R [39].

Thus, XRD and Raman patterns suggest that the carbon microfibers are a disordered arrangement of carbon layers and the TEM micrograph in Figure 4C confirms this conclusion by revealing randomly oriented carbon layers. Some of these layers seem to be larger than the others. As the carbonization temperature increases, these layers tend to become oriented in one direction only. It is also important to mention that the stabilization process to obtain carbon microfibers is crucial because it triggers the oxidation and dehydrogenation of the polymeric nanofibers; oxygen is an initiator for the formation of active centers for cyclization that increases the activation energy and stimulates the dehydrogenation process [29,30]. In this context, SEM micrographs in Figure 4D1,D2) confirm that an adequate temperature (280 °C) had been used for the stabilization process, which avoided the fusion of the microfibers during carbonization.

**Figure 5:**
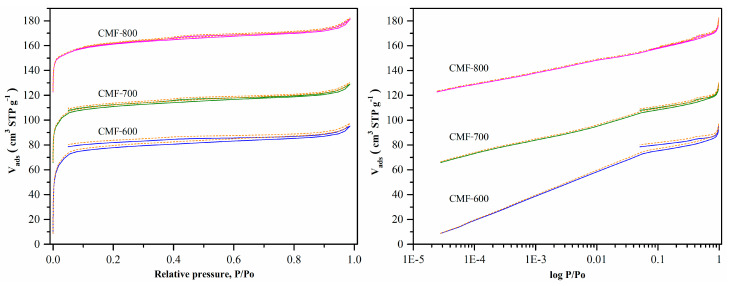
Nitrogen isotherms for carbon materials at −196.15 with equilibration time of 10 min and tolerance 0 (solid line) and 9 (dotted line). Nitrogen adsorption isotherms in linear (left) and logarithmic (right) scale.

Nitrogen adsorption/desorption isotherms at −196.15 are shown in Figure 5. The isotherms showed features of both Type IA (microporous) and Type IV (mesopores), with a narrow hysteresis loop H1, which is typical of slit like larger pores in activated carbons [19]. The hysteresis cycle was subtle, and the high microporosity of the CMFs easily overshadowed it. The high adsorbed volume at a relative pressure of less than 0.1 indicates that the material is mainly microporous. However, the isotherms for CMF-600 and CMF-700 samples presented a particular behavior: the desorption curve does not match the adsorption curve at relative pressures lower than 0.4 (open loop for hysteresis), as did the isotherm of the CMF-800 sample.

To calculate textural parameters such as specific surface area and pore size distribution in the case of microporous materials such as carbons, it is very important to have data at low pressures, starting from 1 × 10^−4^ (relative pressure), because the range considered for BET calculations in mesoporous materials (0.05 to 0.35) does not strictly apply. Figure 5 shows the nitrogen isotherms at an equilibration time of 10 min and tolerance of 0 (±0.001 bar) and 9 (±0.01 bar). Note that the tolerance had no significant effect on the results shown for 0 (solid line) and 9 (dotted line). The one aspect not readily visible in the isotherm, but the acquisition time for these isotherms is shown in Table S1. For the CMF-600 material, regardless of the programmed conditions (equilibrium time: 10 min and tolerance: 0 or 9), it took more than one hour to collect the first point and approximately 71 h to reach a relative pressure of 0.3 (81 h for a complete analysis). Beyond this relative pressure, the time it took to measure the rest of the isotherm was roughly equivalent to the 10 min program, so this isotherm took around three days to be completed. Further increasing the equilibration time, which means that the analysis takes more than three days, was impractical because the liquid nitrogen in the Dewar Flask (P/N 01879 9371) only lasts for four days. Hence, to evaluate the effect of the equilibration time on the hysteresis cycle, isotherms were obtained starting with a relative pressure of 1 × 10^−2^. These isotherms probably cannot be considered to calculate textural parameters, but it will be possible to observe the impact that the equilibration time would have on the hysteresis cycle.

**Figure 6:**
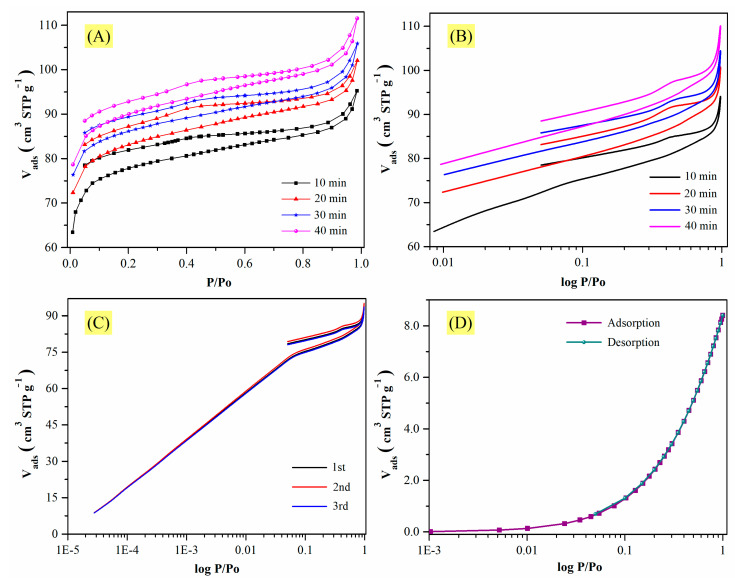
Nitrogen isotherms of CMF-600. At −196 °C and different equilibration times in (**A**) linear and (**B**) logarithmic scales. (**C**) It was repeated 3 times with an equilibration time of 10 min at −196 °C. (**D**) Equilibration time: 10 min at 0 °C.

In Figure 6A,B, the isotherms for CMF-600 are shown considering a tolerance of 0 (±0.001 bar) and equilibration times of 10, 20, 30, and 40 min, starting at a relative pressure of 1 × 10^−2^. Note that there was a remarkable variation in the amounts adsorbed. As the equilibration time increased, so did the adsorbed volume. However, the trend of the hysteresis cycle remained the same, i.e., there was no closure between the adsorption curve and the desorption curve, although the gap decreased. For example, at a lower equilibrium time, at a relative pressure of 0.4, there was a difference of ~4.00 cm^3^ (STP) g^−1^, which decreased to ~3.00 cm^3^ (STP) g^−1^ when the equilibrium time increased, but not enough to close the hysteresis loop. Considering the equilibration time of 10, 20, 30, and 40 min and 89, 95, 98, and 104 cm^3^ STP g^−1^ as the amount adsorbed at a relative pressure of 0.95, respectively, the Pearson’s correlation coefficient was calculated. The value obtained was 0.9922, which means that both variables are strongly related, i.e., the larger the equilibrium time, the greater the adsorbed volume. Therefore, even though the equilibration time does have an impact on the N_2_ uptake, it does not seem to be the only cause of the unclosed hysteresis loop.

The observed variations could also be due to poor material stability, and to verify this hypothesis the same sample underwent a triplicate analysis. Before each isotherm, the material was degassed at 200 °C for 6 h. The obtained isotherms are illustrated in Figure 6C, and they show excellent reproducibility; therefore, the material is not only stable but also regenerable. Additionally, Figure 6D shows a nitrogen adsorption isotherm for the same material (CMF-600) at 0 °C, where the closing of the hysteresis loop can be observed. So, in fact, the analysis temperature seems to be one of the most important factors influencing hysteresis closure. However, the purpose of this study is to explain why, at −196 °C, materials calcined at temperatures below 800 °C exhibit an open hysteresis loop. 

The CMF-700 material had a similar behavior to CMF-600; although it seems that the desorption curve is closer to the adsorption curve, the gap between them remains (Figure 5). The analysis conditions were the same (equilibration time: 10 min and tolerance: 0 and 9). Compared to CMF-600, the acquisition time decreased by 15 h since it took 56 h to reach the relative pressure of 0.3 and 70 h for the complete analysis (Table S1). On the contrary, CMF-800 shows a closure between both curves (adsorption and desorption). The acquisition time is the time programmed for the analysis, with 25 h for the whole analysis (Table S1). Since the detailed study of the CMF-600 material showed that the gap between adsorption and desorption branches persisted, regardless of the equilibration time, and the gap decreased for increasing carbonization temperatures, it is plausible to assume that the chemical composition of the samples may have a determining role in this phenomenon. Adsorption at low pressures for these materials takes place in the micropores, where diffusion is usually hindered. Yet, the material with the most significant micropore volume is CMF-800, which had no problems during the analysis.

In addition to the equilibration time during the analysis, the chemical composition (density of functional groups) could also be considered. Chemical interactions between adsorbate and adsorbent are ruled out, because nitrogen is a nonpolar molecule, and the analysis was carried out at −196.15 °C. On the other hand, it is possible that a higher content of nitrogen and oxygen functional groups within the structure of the carbon layers may lead to pore blocking, especially in small pores. As a matter of fact, the concentration of such functional groups is higher for samples carbonized at lower temperatures and carbon layers are more disordered (as confirmed by TEM). This may be the cause of the results shown in Table S1, where a longer equilibration time is required for isotherm points at low pressures to achieve stability between equilibration time and tolerance. Having said that, it is plausible to propose that the chemical composition of the samples (directly related to the carbonization temperature) has an impact on the atypical behavior of the hysteresis loop for the CMF-600 and CMF-700 materials, which may be further confirmed with enthalpy estimations and ab initio calculations.

**Figure 7:**
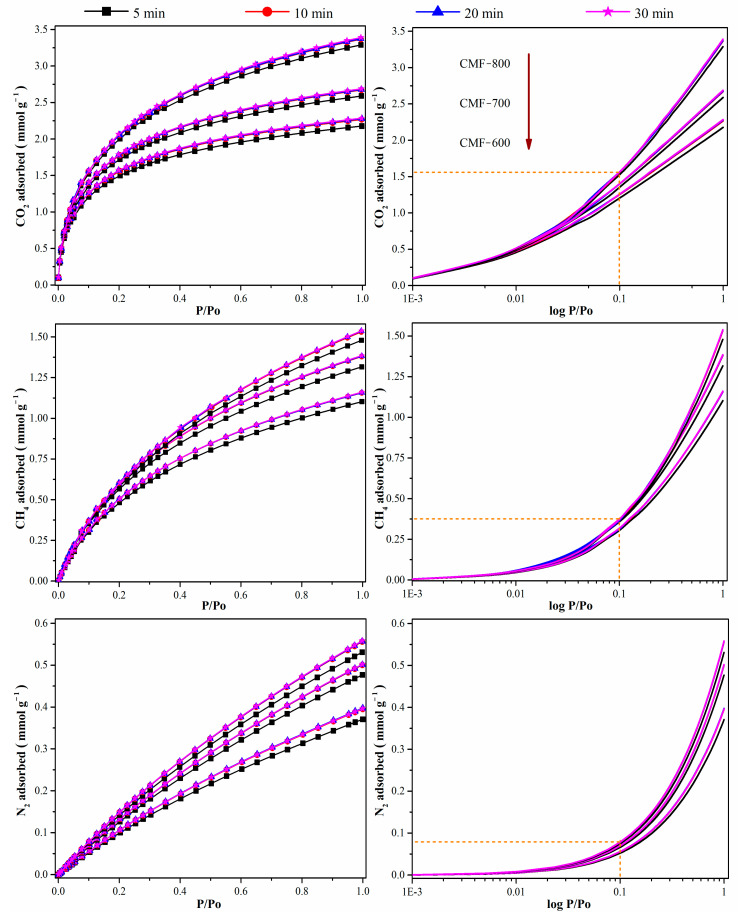
CO_2_, CH_4_, and N_2_ isotherms at 0 °C with equilibration time of 5, 10, 20 and 30 min.

The impact of the equilibration time (5, 10, 20, and 30 min) of other molecules, such as CO_2_, CH_4_, and N_2_ isotherms, at 0 °C on the CMFs is illustrated in Figure 7. Unlike the nitrogen isotherms at −196 °C, the adsorption isotherms for CO_2_, CH_4_, and N_2_ at 0 °C reached stability after an equilibration time of 10 min, regardless of the carbonization temperature of the carbon microfibers. The correlation coefficient between the maximum amount adsorbed at 1 bar and the equilibration time was ~0.8, which is far from 1.0. Therefore, if the measurements are performed at higher temperatures, these two variables (equilibration time at uptake a 1 bar) are independent form one another. The plots on the right side in Figure 7 also show the preference of the CMFs towards CO_2_, which would make it a promising material for CO_2_ selectivity over CH_4_ or N_2_, where microporosity is one of the important parameters that improve selectivity [40].

**Figure 8:**
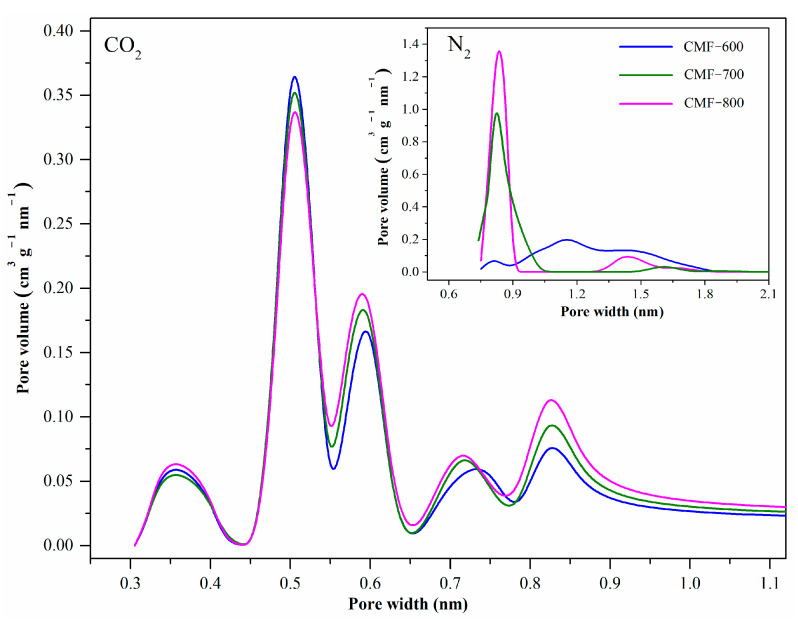
PSD of CO_2_ (0 °C) and N_2_ (−196.15 °C) for carbon microfibers.

From the N_2_ isotherms at −196.15 °C and CO_2_ isotherms at 0 °C, some textural properties have been calculated that allow a better evaluation of the CMFs. The values of these properties are detailed in Table 2 and the average pore size (pore size distribution, PSD) in Figure 8. To calculate the PSD, it is commonly recommended to use the desorption curve; however, due to the behavior they were showing, some authors recommended using the adsorption curve for average pore size calculations [41]. Additionally, in the case of microporous carbon materials, it had also been recommended to calculate the PSD using the CO_2_ isotherms at 0 °C, because the diffusion is much faster and pores as small as 0.4 nm can be accessed [19]. Figure 8 shows the pore size distributions (PSD) of CMFs by both N_2_ isotherms at −196.15 °C and CO_2_ isotherms at 0 °C. The results were obtained directly from the Quantachrome software, considering the NLFDT equilibrium model and slit pores for carbon materials. The nitrogen isotherms are only able to “sense” micropores down to approximately 0.8 nm. The CO_2_ isotherms provide information of micropores down to 0.35 nm, even if there are a small fraction of pores with this diameter, comparable to the results from XRD (0.360 nm). The PSDs obtained from CO_2_ isotherms showed five peaks. Considering the more pronounced peak, centered at 0.50 nm, C-600 shows the greatest pore volume. For the other average sizes (0.60, 0.75 and 0.85 nm), the order is as follows: CMF-800 > CMF-700 > CMF-600, which is consistent with the adsorbed volume of each of the materials (Figure 7). Based on PSDs calculated by CO_2_ isotherms, the CMF samples have supermicropores (2.0 nm > width > 0.7 nm) and ultramicropores (width < 0.7 nm) [19,42].

From the results in Table 2, considering N_2_ adsorption at −196.15 °C, the following observations can be made: (i) the specific surface area (by BET equation) increases from 296 m^2^ g^−1^ (CMF-600) to 635 m^2^ g^−1^ (CMF-800), and with it, the microporosity from 79% to 89%; (ii) the total volume is duplicated between the CMF-600 and CMF-800 material; (iii) the micropore volume increases with increasing carbonization temperature and this behavior is corroborated by three methods (t-plot, α-plot, and DR); (iv) the average pore diameter decreases with increasing calcination temperature and NLDFT and DR models demonstrate that these materials are highly microporous. Considering CO_2_ isotherms, the micropore specific areas (by DR) have been calculated for the three samples and compared to A_BET_(N_2_) (see Table 2). Note that A_BET_(N_2_)/A_DR_(CO_2_) increased for higher carbonization temperatures. Actually, A_DR_(CO_2_) increased moderately with higher carbonization temperatures, whereas A_BET_(N_2_) increased considerably. The average pore diameter remained almost constant, ~1.4 nm by DR model and ~0.5 nm by NLDFT model, which is consistent with the pore size distribution (Figure 8) as calculated from CO_2_ isotherms. Therefore, as carbonization temperature increases in this range (600–800 °C), the specific volume of ultramicropores (as sensed by CO_2_) remains approximately the same, whereas the surface area and volume of micropores larger than 0.7 nm increases significantly. These values suggest that the microporosity cannot be directly related to hysteresis cycle behavior. Three different models (t-plot, alpha-plot, and DR) have been used to determine the microporosity in these materials and all of them confirm that CMF-800 has the largest micropore volume, the largest micropore area and the narrowest pores (as sensed by N_2_ isotherms). Hence, in the specific case of carbon microfibers synthesized from PAN electrospinning, a higher microporosity, per se, does not lead to greater diffusion problems for the nitrogen molecule and should not be the cause of the open hysteresis loop.

To evaluate the influence of the chemical composition, the interaction energy between the different gases (N_2_, CO_2_, and CH_4_) and the carbon microfibers (CMF-600, CMF-700, and CMF-800), two methods will be employed: (i) the isosteric enthalpy of adsorption and (ii) a theoretical analysis by molecular dynamics.

**Figure 9:**
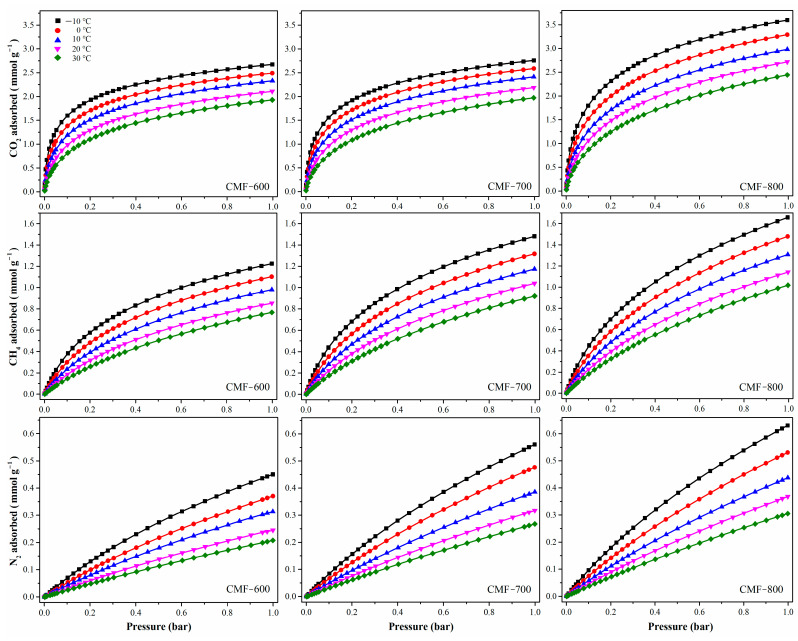
CO_2_, CH_4_, and N_2_ isotherms of CMFs at −10 °C, 0 °C, 10 °C, 20 °C, and 30 °C.

**Figure 10:**
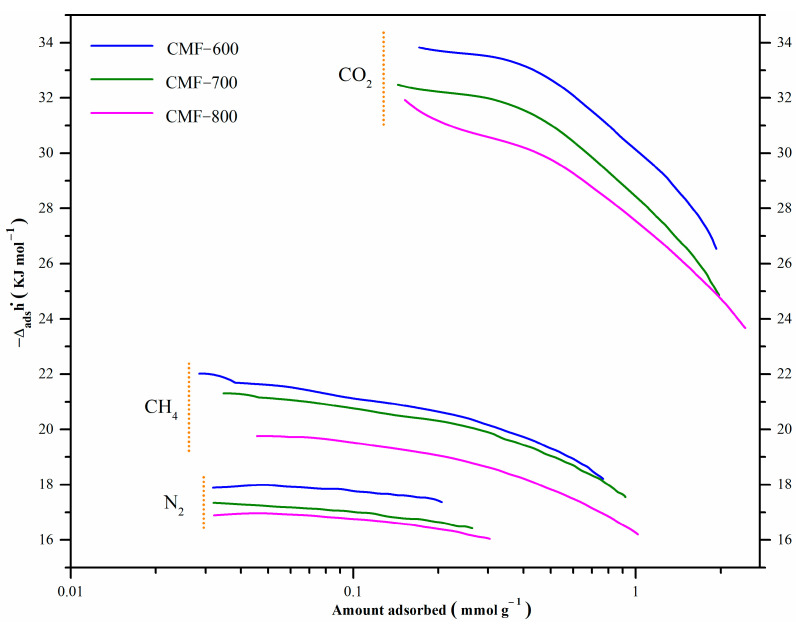
CO_2_, CH_4_, and N_2_ isosteric enthalpy for carbon microfibers.

In this work, the isosteric enthalpy of adsorption has been calculated indirectly, that is, by using adsorption isotherms at different temperatures. It has been reported that when the process is dominated by physisorption, the temperatures selected for the analysis do not have much influence if the difference between them is not larger than 20 °C [43]. Five temperatures have been considered (−10, 0, 10, 20, and 30 °C). The CO_2_, CH_4_, and N_2_ isotherms for CMF-600, CMF-700, and CMF-800 are shown in Figure 9; increments in the carbonization temperature also increase the adsorbed concentrations in all cases, CO_2_, CH_4_, and N_2_ (Table 3). Additionally, all materials adsorbed more CO_2_ than CH_4_ and N_2_, particularly in the lower pressure range, which means that there is a higher affinity of the CMFs towards the CO_2_ molecule. Actually, CO_2_ selectivity on all samples (defined as the ratio CO_2_/CH_4_) slightly increased with increasing temperature. Although selectivity tended to decrease with increasing carbonization temperature (and hence less functional groups), it remained above 2 for sample MCF800 regardless of the temperature. This behavior is consistent with the isosteric enthalpies of adsorption of the adsorbate under study, shown in Figure 10. Additionally, Pearson’s correlation coefficient (Table 3) has been calculated in an attempt to correlate the experiment temperature with (i) the amount adsorbed of each gas and (ii) CO_2_ selectivity. In the first case, all values are close to −0.99; the negative value means that the variables are inversely related, i.e., with increasing temperature, the amount adsorbed will decrease. In the case of CO_2_ selectivity, it shows a positive correlation, around 0.95, which means that both variables are directly related, so an increase in temperature also increases selectivity.

The isosteric enthalpy of CO_2_ adsorption at low surface coverage was around −33.8, −32.3, and −31.4 kJmol^−1^ for materials CMF-600, CMF-700, and CMF-800, respectively. These values decrease with an increment in surface coverage, which can be associated with the energy heterogeneity of the carbon microfibers surface adsorption sites. The isosteric enthalpy of adsorption of these materials followed the order: CMF-600 > CMF-700 > CMF-800, so the interaction of CO_2_ with CMF-600 was stronger than with CMF-800, because it released a greater amount of energy. The behavior is similar in the isosteric enthalpy of CH_4_: the material with higher affinity was CMF-600 with −21.6 KJmol^−1^; CMF-700 and CMF-800 with −21.1 KJmol^−1^ and −19.8 KJmol^−1^, respectively. CMF-800 is the material with the highest surface area, highest microporosity, and highest uptakes for both CO_2_ and CH_4_. Sample CMF-800 was the one with the lowest adsorption enthalpies, and therefore the interaction energy between the three probe gas molecules and the sample is lower as compared to the interaction in CMF-600 and CMF-700 materials. Based on these results, it is evident that the isosteric enthalpy of adsorption is not strictly related to the microporosity or specific surface area. Regarding the chemical composition, CMF-800 is the material with the highest carbon content and the lowest content of both nitrogen and oxygen (Table 1 and Figure 1B). Therefore, the presence of functional groups derived from these heteroatoms seems to lead to stronger interactions of these probe molecules in CMF-600 and CMF-700 samples and thus higher adsorption enthalpies, although not the highest uptakes. Furthermore, it is interesting to observe the behavior of the isosteric enthalpies as a function of coverage. For example, in Figure 9, the enthalpy curves for CO_2_ show a pronounced decrease with increasing coverage, which means that the interaction between the CO_2_ molecules with the surface groups of the CMFs is quite strong when the first CO_2_ molecules are adsorbed. On the other hand, the slope of the curves for CH_4_ is less pronounced as a result of a weaker interaction of this gas with the surface of the CMFs samples. In the case of N_2_, there is practically no chemical interaction since the lines show a constant behavior. Therefore, these materials could be potentially used as CO_2_ adsorbents in CO_2_:CH_4_ mixtures or in CO_2_:N_2_ mixtures.

**Figure 11:**
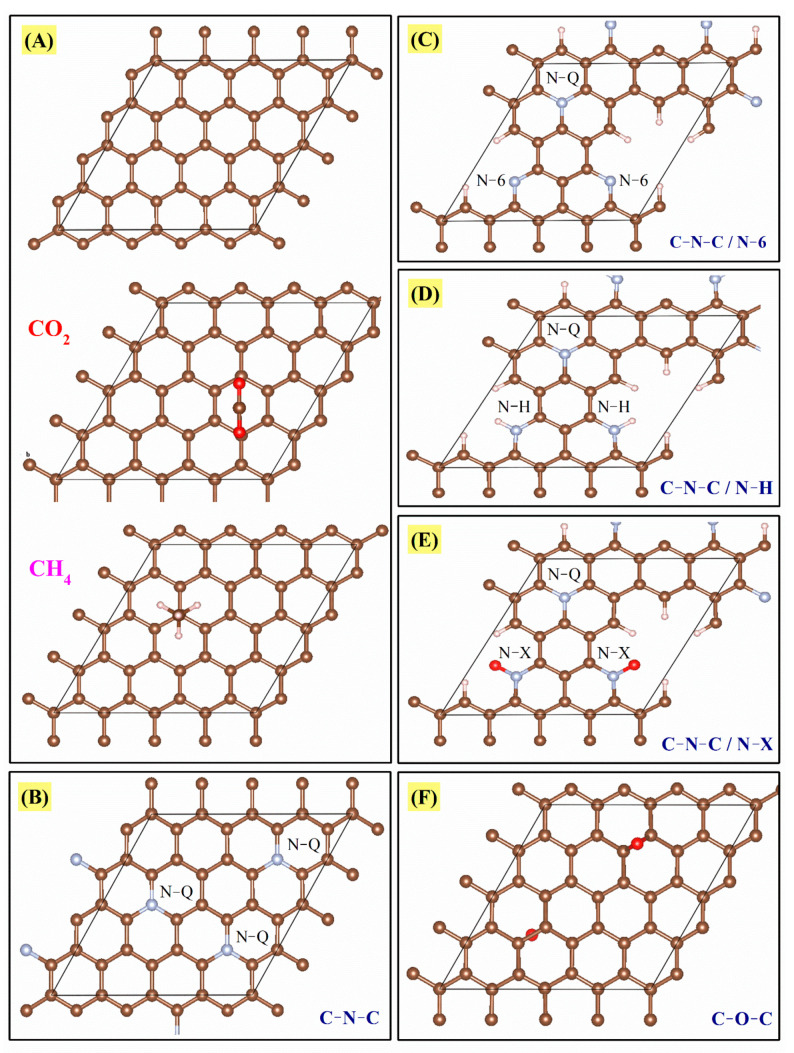
Panel with the configurations proposed for the physisorption of CO_2_ and CH_4_ molecules on the carbon sheet and its different functional groups, (**A**) C=C, (**B**) C-N-C, (**C**) C-N-C and N-6, (**D**) C-N-C and N-H, (**E**) C-N-C and N-X, and (**F**) C-O-C.

In addition to the isosteric enthalpy of adsorption, the binding energies of CO_2_ and CH_4_ with graphitic materials were estimated by ab-initio calculations (periodic DFT, more details in Section 2.4: computational study) in order to understand the possible intermolecular interactions. The CO_2_ and CH_4_ interaction energies were analyzed considering a graphene sheet containing the following groups: (i) pure carbon (C=C), (ii) C-N-C (quaternary-N), (iii) C-N-C and N-6 (pyridine-N), (iv) C-N-C and N-H, (v) C-N-C and N-X (pyridinic-N-oxide), and (vi) C-O-C (ethoxy). These groups will be called A, B, C, D, E, and F, respectively. These arrangements are shown in Figure 11 and the results in Table 4.

This analysis was performed to give a general idea of the interaction between the studied molecules (CO_2_ and CH_4_) and each of the functional groups theoretically present in the CMFs, considering a single carbon sheet; confinement effects and multiple interactions will be addressed elsewhere. CO_2_ and CH_4_ molecules are nonpolar, capable of forming weak interactions with the functional groups in CMFs. All binding energy values in Table 4 show low affinity energies; however, among all functional groups, the oxygen functional groups stand out as having the most negative and highest absolutes. Nitrogen groups (B, C, D, and E) also have an effect, though one much more modest. Finally, A (pure carbon) and B (quaternary-N) are the configurations with lower reactivity. This fact means that a chemical surface rich in oxygen is preferred to enhance CO_2_ adsorption. For CH_4_, a similar behavior occurs; the F configuration shows the highest reactivity (C-O-C bonds). After the functional groups with oxygen, the most reactive species are N-6 (pyridine-N) and N-H. These calculations agree with experimental results; for instance, the CMF-600 material presents a higher amount of oxygen (Figure 1B and Table 1) and, therefore, a higher binding energy (Table 4). In Figure 10 this material has the highest isosteric enthalpy of adsorption, i.e., the higher interaction energy between the adsorbates (CO_2_ or CH_4_) and adsorbent (CMFs). At this point, N_2_ adsorption was not calculated due to the very low uptake and adsorbate–adsorbent interaction. Moreover, CH_4_ and CO_2_ provides the specific reactivity trends of positive (H end) and negative (O end) molecules, respectively.

In this context, some studies have already focused on comparing the π-π type Van der Waals interactions of CO_2_ with: (i) benzene, pyridine, and pyrrole [44,45] or (ii) carbonyl compounds [46]. And the CH/π Interactions of CH_4_ with (i) benzene [47,48,49] or (ii) pyridine [50]. In general, they have shown an interaction between CO_2_ and CH_4_ molecules with the different functional groups, and in some cases, a weak interaction, consistent with the results shown here. It is worth highlighting that the simulations presented herein consider a single layer of grapheme. While other effects, such as confinement, multiple sites and functional groups, could have further impact on the results, they are beyond the scope of the present manuscript and are currently subject of follow up work.

Although all the binding energy values (calculated in this work) are relatively small, they allowed concluding that the presence of these functional groups favors the interaction with molecules such as CO_2_ or CH_4_. Furthermore, the amount of these species has a crucial role, which likely caused the samples CMF-600 and CMF-700 to show the following behaviors: (i) the N_2_ isotherms (at −196.15 °C) did not present a closed hysteresis cycle and (ii) the CO_2_ and CH_4_ adsorption uptakes were lower as compared to CMF-800 (which had the highest C and lowest N/O content of all MCF samples). An unclosed hysteresis loop in N_2_ adsorption for carbonized materials at low temperatures may be a synergistic behavior between chemical composition and textural properties because these materials are the ones that present a lower degree of graphitization and more significant disorder in the arrangement of carbon sheets (behavior observed by Raman and XRD). Another conclusion that can be drawn is that the increase in carbonization temperature leads to a higher degree of graphitization, this means, for example, that temperatures above 2000 °C are only going to present C-C and C=C bonds (configuration A), and based on the results in Table 3, these materials are going to adsorb a lower amount of CO_2_, due to the low or null interaction between the CO_2_ molecule and the sp^2^ and sp^3^ carbons (Table 3 and A arrangement in Figure 11); for this reason, it is imperative to choose a temperature that allows preserving a concentration of functional groups for an efficient adsorption of CO_2_ or CH_4_, because an increase in carbonization temperature also raises the specific surface area. Still, surface chemistry dominates the adsorption phenomena in these materials.

## 4. Conclusions

The main purpose of this work was to explore the effect of carbonization temperature and chemical composition of PAN-derived carbon microfibers on N_2_, CO_2_, and CH_4_ equilibration time and adsorption. In particular, we tried to understand why the nitrogen isotherms at −196.15 °C for samples carbonized at temperatures below 800 °C show an open hysteresis loop. At −196.15 °C, an open hysteresis loop persists in the CMFs samples carbonized at 600 and 700 °C, despite extending the equilibration time from 10 min to 40 min, which disappears at higher temperatures (e.g., 0 °C). By XRD, Raman, and TEM, it was demonstrated that these materials have a low ordering of carbon layers. Moreover, the distinct surface composition of the CMFs samples may also play a role. Higher adsorption enthalpies, and thus stronger adsorbent/adsorbate interaction energies, were observed experimentally and estimated by ab initio calculations for the samples calcined at lower temperatures (CMF-600 and CMF-700) considering three probe gases (N_2_, CO_2_, and CH_4_). These materials have a higher number of heteroatoms (oxygen and nitrogen functional groups), so chemical composition and structural disorder are likely to be the main reasons for the unclosed hysteresis loop in the nitrogen isotherms at −196.15 °C. CMF-600 (with O/N ratio of 0.37) shows the highest adsorbent/adsorbate interaction energies, although it has the lowest specific surface area. When the O/N ratio increases to 0.39 and 0.67—for CMF-700 and CMF-800, respectively—the interaction energy decreases. By comparing the three probe molecules, CMFs show a high affinity for the CO_2_ molecule with respect to CH_4_ and N_2_ molecules, regardless of the carbonization temperature, which entitles these materials as selective CO_2_ adsorbents for separation from gas mixtures containing CH_4_ or N_2_. The carbonization temperature of 800 °C is recommended, which leads to materials with a suitable porous texture and low content of surface heteroatoms, favoring high CO_2_ uptake while keeping acceptable selectivity with regards to CH_4_/N_2_ and requiring moderate adsorption enthalpies for desorption.

## Figures and Tables

**Table 1:** Structural and chemical properties of CMFs.

		CMF-600	CMF-700	CMF-800
XRD	2θ (°)	24.68	24.77	24.87
	d_002_ (nm)	0.360	0.359	0.358
	2θ (°)	43.34	43.39	43.45
	d_100_ (nm)	0.208	0.208	0.208
	FWHM_(002)_ (2θ)	10.025	9.440	8.380
	$L_{c\left(002\right)}$ (nm)	0.820	0.871	0.981
	FWHM_(100)_ (2θ)	4.870	4.740	4.130
	$L_{a\left(100\right)}$ (nm)	3.587	3.686	4.231
Raman [13]	R = I_D_/I_G_	1.62	1.52	1.26
	$L_{a}=4.4/R$	2.71	2.88	3.47
XPS [13]	C 1s (% *w/w*)	78.0	82.0	85.0
	N 1s (% *w/w*)	16.0	13.0	9.0
	O 1s (% *w/w*)	6.0	5.0	6.0

**Table 2:** Textural properties of CMFs by N_2_ at −196.15 °C and CO_2_ at 0 °C.

		CMF-600	CMF-700	CMF-800
N_2_ adsorption	A_BET_ (m^2^ g^−^^1^)	296	431	635
	A_MICRO (t)_ (m^2^ g^−^^1^)	266	398	597
	V_T_ (cm^3^ g^−^^1^)	0.14	0.19	0.27
	V_MICRO (t)_ (cm^3^ g^−^^1^)	0.11	0.16	0.24
	V_MESO_ (cm^3^ g^−^^1^)	0.03	0.03	0.03
	% M	79.0	84.0	89.0
	V_MICRO (α)_ (cm^3^ g^−^^1^)	0.10	0.15	0.23
	V_MICRO (DR)_ (cm^3^ g^−^^1^)	0.13	0.17	0.25
	D_DR_ (nm)	2.05	1.43	1.03
	D_NLDFT_ (nm)	1.15	0.80	0.80
CO_2_ adsorption	A_DR_ (m^2^ g^−^^1^)	215	233	272
	V_T_ (cm^3^ g^−^^1^)	0.10	0.11	0.15
	V_MICRO (DR)_ (cm^3^ g^−^^1^)	0.07	0.08	0.09
	D_DR_ (nm)	1.40	1.43	1.48
	D_NLDFT_ (nm)	0.50	0.50	0.50
N_2_ and CO_2_	A_BET_ (N_2_)/A_DR_ (CO_2_)	1.38	1.85	2.33

A_BET_ = specific surface area by BET equation; A_MICRO (t)_ = microporous area by t-model; V_T_ = total volume at 0.95 in relative pressure; V_MICRO (t)_ = microporous volume by t-model; V_MESO_ = mesoporous volume; % M = % microporosity calculated using total volume and micropore volume (t-model) V_MICRO (α)_ = microporous volume by α-plot; V_MICRO (DR)_ = microporous volume by Dubinin–Radushkevich; D_DR_ = average pore diameter by Dubinin–Radushkevich; D_NLDFT_ = average pore diameter by NLDFT model; A_DR_ = microporous area by Dubinin–Radushkevich.

**Table 3:** CO_2_, CH_4_, and N_2_ adsorbed (mmol/g adsorbent) at 1.0 bar in PANMF carbonized.

T (°C)	CMF-600				CMF-700				CMF-800			
	CO_2_	CH_4_	N_2_	CO_2_/CH_4_	CO_2_	CH_4_	N_2_	CO_2_/CH_4_	CO_2_	CH_4_	N_2_	CO_2_/CH_4_
−10	2.68	1.22	0.45	2.20	2.76	1.48	0.56	1.89	3.60	1.66	0.63	2.17
0	2.49	1.10	0.37	2.36	2.59	1.32	0.48	1.96	3.29	1.48	0.53	2.22
10	2.33	0.98	0.31	2.38	2.41	1.17	0.39	2.06	2.98	1.31	0.44	2.27
20	2.11	0.85	0.25	2.48	2.19	1.04	0.32	2.11	2.72	1.14	0.37	2.39
30	1.93	0.77	0.21	2.51	1.97	0.92	0.27	2.14	2.44	1.02	0.31	2.39
r	−0.9991	−0.9975	−0.9934	0.9592	−0.9981	−0.9982	−0.9949	0.9813	−0.9993	−0.9977	−0.9944	0.9684

r = Pearson correlation coefficient.

**Table 4:** Binding energy between CMFs and gases (CO_2_ and CH_4_).

Name	Functional Groups	CO_2_/E (eV)	CH_4_/E (eV)
A	C=C	0.008	−0.021
B	C-N-C	0.020	0.021
		0.052	0.019
C	C-N-C and N-6	−0.032	0.015
		0.037	0.030
D	C-N-C and N-H	−0.021	−0.012
		0.001	−0.024
E	C-N-C and N-X	−0.019	−0.006
		−0.078	−0.018
F	C-O-C	−0.208	−0.191
		−0.184	−0.175

## Data Availability

Not applicable.

## Supplementary Materials

The following are available online at https://www.mdpi.com/article/10.3390/ma14143914/s1, Table S1: Adsorption data for CMFs carbonized at 600, 700, and 800 °C.

## References

[1] Bazan-Wozniak A., Nowicki P., Pietrzak R. (2021). Removal of NO_2_ from gas stream by activated bio-carbons from physical activation of residue of supercritical extraction of hops. Chem. Eng. Res. Des..

[2] Ishita I., Singhal R. (2020). Porous multi-channel carbon nanofiber electrodes using discarded polystyrene foam as sacrificial material for high-performance supercapacitors. J. Appl. Electrochem..

[3] Othman F.E.C., Yusof N., Harun N.Y., Bilad M.R., Jaafar J., Aziz F., Salleh W.N.W., Ismail A.F. (2020). Novel activated carbon nanofibers composited with cost-effective graphene-based materials for enhanced adsorption performance toward methane. Polymers.

[4] Thaveemas P., Chuenchom L., Techasakul S., Watcharin W., Dechtrirat D. (2020). Facile preparation of magnetic carbon nanofiber composite from nata de coco for removal of methylene blue dye from water. IOP Conf. Ser. Mater. Sci. Eng..

[5] Gregg S.J., Sing K.S.W. (1982). Adsorption, Surface Area and Porosity.

[6] Qi L., Tang X., Wang Z., Peng X. (2017). Pore characterization of different types of coal from coal and gas outburst disaster sites using low temperature nitrogen adsorption approach. Int. J. Min. Sci. Technol..

[7] Thommes M., Cychosz K.A. (2014). Physical adsorption characterization of nanoporous materials: Progress and challenges. Adsorption.

[8] Terzyk A.P., Rychlicki G. (1999). Calorimetric investigations of molecular interactions in the adsorbate/microporous activated carbon system. Towards the mechanism of adsorption in micropores. Adsorpt. Sci. Technol..

[9] Wu H., Thibault C.G., Wang H., Cychosz K.A., Thommes M., Li J. (2016). Effect of temperature on hydrogen and carbon dioxide adsorption hysteresis in an ultramicroporous MOF. Microporous Mesoporous Mater..

[10] Sircar S., Wu H., Li J., Lueking A.D. (2011). Effect of time, temperature, and kinetics on the hysteretic adsorption-desorption of H_2_, Ar, and N_2_ in the metal-organic framework Zn_2_(bpdc)_2_(bpee). Langmuir.

[11] Stoeckli F. (1998). Recent developments in Dubinin’s Theory. Carbon N. Y..

[12] Quantachrome I. (2015). Characterizing Porous Materials and Powders: Gas Sorption System Operating Manual.

[13] Ojeda-López R., Ramos-Sánchez G., Esparza-Schulz J.M., Lartundo L., Domínguez-Ortiz A. (2017). On site formation of N-doped carbon nanofibers, an efficient electrocatalyst for fuel cell applications. Int. J. Hydrogen Energy.

[14] Laffont L., Monthioux M., Serin V., Mathur R.B., Guimon C., Guimon M.F. (2004). An EELS study of the structural and chemical transformation of PAN polymer to solid carbon. Carbon N. Y..

[15] Kumar A., Ganguly A., Papakonstantinou P. (2012). Thermal stability study of nitrogen functionalities in a graphene network. J. Phys. Condens. Matter.

[16] Gu S.Y., Ren J., Wu Q.L. (2005). Preparation and structures of electrospun PAN nanofibers as a precursor of carbon nanofibers. Synth. Met..

[17] Ojeda-López R., Esparza-Schulz J.M., Pérez-Hermosillo I.J., Hernández-Gordillo A., Domínguez-Ortiz A. (2019). Improve in CO_2_ and CH_4_ Adsorption Capacity on Carbon Microfibers Synthesized by Electrospinning of PAN. Fibers.

[18] Brunauer S., Emmett P.H., Teller E. (1938). Gases in Multimolecular Layers. J. Am. Chem. Soc..

[19] Thommes M., Kaneko K., Neimark A.V., Olivier J.P., Rodriguez-Reinoso F., Rouquerol J., Sing K.S.W. (2015). Physisorption of gases, with special reference to the evaluation of surface area and pore size distribution (IUPAC Technical Report). Pure Appl. Chem..

[20] Rouquerol J., Llewellyn P., Rouquerol F. (2007). Is the BET equation applicable to microporous adsorbents?. Stud. Surf. Sci. Catal..

[21] Ojeda-López R., Aguilar-Huerta E., Maia D.A.S., Azevedo D.C.S., Felipe C., Domíngez-Ortiz A. (2020). Tailoring synthesis conditions of carbon microfibers to enhance the microporosity, CO_2_ and CH_4_ adsorption by using the response surface methodology. Microporous Mesoporous Mater..

[22] Kresse G., Furthmuller J. (1996). Efficient iterative schemes for ab initio total-energy calculations using a plane-wave basis set. Phys. Rev. B.

[23] Blöchl P.E. (1994). Projector augmented-wave method. Phys. Rev. B.

[24] Valero R., Gomes J.R.B., Truhlar D.G., Illas F. (2010). Density functional study of CO and NO adsorption on Ni-doped MgO (100). J. Chem. Phys..

[25] Ouyang Q., Cheng L., Wang H., Li K. (2008). Mechanism and kinetics of the stabilization reactions of itaconic acid-modified polyacrylonitrile. Polym. Degrad. Stab..

[26] Dalton S., Heatley F., Budd P.M. (1999). Thermal stabilization of polyacrylonitrile fibres. Polymer.

[27] Wangxi Z., Jie L., Gang W. (2003). Evolution of structure and properties of PAN precursors during their conversion to carbon fibers. Carbon N. Y..

[28] Xue Y., Liu J., Liang J. (2013). Correlative study of critical reactions in polyacrylonitrile based carbon fiber precursors during thermal-oxidative stabilization. Polym. Degrad. Stab..

[29] Rahaman M.S.A., Ismail A.F., Mustafa A. (2007). A review of heat treatment on polyacrylonitrile fiber. Polym. Degrad. Stab..

[30] Fitzer E., Müller D.J. (1975). The influence of oxygen on the chemical reactions during stabilization of pan as carbon fiber precursor. Carbon N. Y..

[31] Almuhamed S., Bonne M., Khenoussi N., Brendle J., Schacher L., Lebeau B., Adolphe D.C. (2016). Electrospinning composite nanofibers of polyacrylonitrile/synthetic Na-montmorillonite. J. Ind. Eng. Chem..

[32] Kim C., Park S.H., Cho J.I., Lee D.Y., Park T.J., Lee W.J., Yang K.S. (2004). Raman spectroscopic evaluation of polyacrylonitrile-based carbon nanofibers prepared by electrospinning. J. Raman Spectrosc..

[33] Zussman E., Chen X., Ding W., Calabri L., Dikin D.A., Quintana J.P., Ruoff R.S. (2005). Mechanical and structural characterization of electrospun PAN-derived carbon nanofibers. Carbon N. Y..

[34] Ladd M., Palmer R. (2013). Structure Determination by X-ray Crystallography: Analysis by X-rays and Neutrons.

[35] Cullity B.D., Stock S.R. (2014). Elements of X-ray Diffraction.

[36] Thamer B.M., El-Hamshary H., Al-Deyab S.S., El-Newehy M.H. (2019). Functionalized electrospun carbon nanofibers for removal of cationic dye. Arab. J. Chem..

[37] Inagaki M., Kang F., Inagaki M., Kang F. (2016). Materials Science and Enginnering of Carbon: Characterization.

[38] Wang Y., Serrano S., Santiago-Avilés J.J. (2003). Raman characterization of carbon nanofibers prepared using electrospinning. Synth. Met..

[39] Andrei R.D., Marinoiu A., Marin E., Enache S., Carcadea E. (2020). Carbon nanofibers production via the electrospinning process. Energies.

[40] Moura P.A.S., Vilarrasa-Garcia E., Maia D.A.S., Bastos-Neto M., Ania C.O., Parra J.B., Azevedo D.C.S. (2018). Assessing the potential of nanoporous carbon adsorbents from polyethylene terephthalate (PET) to separate CO_2_ from flue gas. Adsorption.

[41] Groen J.C., Peffer L.A.A., Pérez-Ramírez J. (2003). Pore size determination in modified micro- and mesoporous materials. Pitfalls and limitations in gas adsorption data analysis. Microporous Mesoporous Mater..

[42] Kaneko K., Ishii C. (1992). Superhigh surface area determination of microporous solids. Colloids Surf..

[43] Ojeda-López R., Domínguez-Ortiz A., Felipe C., Cervantes-Uribe A., Pérez-hermosillo I.J., Esparza-schulz J.M. (2021). Isosteric Enthalpy Behavior of CO_2_ Adsorption on Micro-Mesoporous Materials: Carbon Microfibers (CMFs), SBA-15, and Amine-Functionalized SBA-15. J. Compos. Sci..

[44] Chen L., Cao F., Sun H. (2013). Ab initio study of the π-π Interactions between CO_2_ and benzene, pyridine, and pyrrole. Int. J. Quantum Chem..

[45] Lee H.M., Youn I.S., Saleh M., Lee J.W., Kim K.S. (2015). Interactions of CO_2_ with various functional molecules. Phys. Chem. Chem. Phys..

[46] Wang J., Wang M., Hao J., Fujita S.I., Arai M., Wu Z., Zhao F. (2010). Theoretical study on interaction between CO_2_ and carbonyl compounds: Influence of CO_2_ on infrared spectroscopy and activity of C=O. J. Supercrit. Fluids.

[47] Albertí M., Aguilar A., Lucas J.M., Pirani F. (2012). Competitive role of CH_4_-CH_4_ and CH-π interactions in C_6_H_6_-(CH_4_)_n_ Aggregates: The transition from dimer to cluster features. J. Phys. Chem. A.

[48] Ringer A.L., Figgs M.S., Sinnokrot M.O., Sherrill C.D. (2006). Aliphatic C-H/π Interactions: Methane-Benzene, Methane-Phenol, and Methane-Indole Complexes. J. Phys. Chem. A.

[49] Tsuzuki S., Honda K., Uchimaru T., Mikami M., Tanabe K. (2000). The magnitude of the CH/π interaction between benzene and some model hydrocarbons. J. Am. Chem. Soc..

[50] Gou Q., Spada L., Vallejo-López M., Lesarri A., Cocinero E.J., Caminati W. (2014). Interactions between alkanes and aromatic molecules: A rotational study of pyridine-methane. Phys. Chem. Chem. Phys..

