# Supplementary material for: Effect of Calcination Temperature and Chemical Composition of PAN-Derived Carbon Microfibers on N2, CO2, and CH4 Adsorption
Source: Materials (Basel). 2021 Jul 13;14(14):3914. doi: 10.3390/ma14143914 (PMC8305112; doi:10.3390/ma14143914)
Supplement: Supplementary file 1 [file materials-14-03914-s001.zip › materials-1223510-supplementary.pdf]

materials

MDPI

Supplementary material

Effect of Calcination Temperature and Chemical Composition of PAN-Derived Carbon Microfibers on N_2, CO_2, and CH_4 Adsorption

Reyna Ojeda-López [1,2,*], Guadalupe Ramos-Sánchez [2,3], Cinthia García-Mendoza [4], Diana C. S. Azevedo [1], Ariel Guzmán-Vargas [5] and Carlos Felipe [6,*]

[1] Laboratório de Pesquisa em Adsorção e Captura de CO_2 ($LPACO_2$), Departamento de Engenharia Química, Universidade Federal do Ceará (UFC), Fortaleza 60455-760, CE, Brazil; diana@gpsa.ufc.br
[2] Departamento de Química, Universidad Autónoma Metropolitana-Iztapalapa (UAM-I), 09340 Mexico City, Mexico; gramossa@conacyt.mx
[3] CONACYT, Universidad Autónoma Metropolitana-Iztapalapa (UAM-I), 09340 Mexico City, Mexico
[4] Laboratorio de Nanotecnología, Centro de Investigación de Ciencia y Tecnología Avanzada de Tabasco (CICTAT), División Académica de Ingeniería y Arquitectura, Universidad Juárez Autónoma de Tabasco (UJAT), 86690 Tabasco, Mexico; cinthia.garcia@ujat.mx
[5] Laboratorio de Investigación en Materiales Porosos, Catálisis Ambiental y Química Fina, ESIQIE, Instituto Politécnico Nacional (IPN), 07738 Mexico City, Mexico; aguzmanv@ipn.mx
[6] Departamento de Biociencias e Ingeniería, Centro Interdisciplinario de Investigaciones y Estudios sobre Medio Ambiente y Desarrollo (CIIEMAD), Instituto Politécnico Nacional (IPN), 07340 Mexico City, Mexico
* Correspondence: rol@xanum.uam.mx (R.O.-L.); cfelipe@ipn.mx (C.F.)

Table S1. Adsorption data for CMFs carbonized at 600, 700, and 800 °C.

CMF-600			CMF-700			CMF-800		
Relative pressure	Volume (STP) cc	Time (min)	Relative pressure	Volume (STP) cc	Time (min)	Relative pressure	Volume (STP) cc	Time (min)
2.78E-05	8.70370	1147.4	2.80E-05	65.8531	640.3	2.44E-05	122.7717	93.1
5.65E-05	13.9942	1356.8	4.47E-05	68.6694	1063.5	5.61E-05	126.5486	251.5
8.19E-05	17.5764	1831.8	6.76E-05	70.9139	1566.3	7.16E-05	127.5458	304.4
1.30E-04	21.3593	1907.2	1.20E-04	74.1724	1717.1	9.54E-05	128.5430	407.8
1.98E-04	24.7655	1939.9	2.43E-04	77.7294	1844.9	2.38E-04	132.0558	482.3
3.13E-04	28.5493	2118.1	4.76E-04	80.6591	1954.5	4.79E-04	134.7755	600.9
4.94E-04	32.7110	2560.2	7.23E-04	82.4226	2099.9	7.17E-04	136.3846	686.6
9.75E-04	38.5220	3049.8	9.63E-04	83.7847	2239.8	9.56E-04	137.8244	728.2
0.00268	47.0430	3173.8	0.00264	88.0817	2364.2	0.00236	141.6129	754.7
0.00458	51.6872	3351.4	0.00511	91.3303	2486.7	0.00486	145.0538	770.4
0.00857	57.0125	3457.3	0.00758	93.4119	2503.3	0.01046	148.4946	785.1
0.01840	63.4748	3507.7	0.00965	94.7718	2622.2	0.02149	150.7527	796.6
0.03687	69.1935	3721.1	0.02887	101.8279	2753.9	0.04830	153.9277	807.9
0.05552	72.8189	3773.5	0.05456	106.1074	2863.6	0.07477	156.3188	818.2
0.07693	74.4595	3824.8	0.08027	107.4922	2974.2	0.11187	158.3144	828.5
0.10351	75.4613	3876.1	0.10396	108.4307	3083.7	0.12845	159.0019	839.2
0.13051	76.2203	3927.4	0.13131	109.2889	3125.3	0.15916	160.0259	849.9
0.15475	76.8431	3978.7	0.15455	109.9160	3176.7	0.17932	160.6144	860.6
0.17824	77.3682	4030.6	0.17856	110.4848	3363.2	0.20468	161.2593	871.3

0.20224	77.8417	4081.3	0.20209	110.9840	3374.2	0.22755	161.7886	882.5
0.22667	78.2856	4132.6	0.22657	111.4573	3385.7	0.25176	162.3028	892.7
0.25132	78.6881	4183.9	0.25137	111.8992	3396.6	0.27615	162.7974	903.4
0.27601	79.0697	4251.4	0.27602	112.3102	3407.1	0.30115	163.2479	914.1
0.30084	79.4285	4271.1	0.30072	112.7015	3419.8	0.34934	164.0340	924.8
0.34946	80.0226	4281.5	0.34998	113.3976	3420.3	0.40041	164.8089	935.5
0.39974	80.6158	4292.1	0.39909	114.0630	3431.2	0.42442	165.1694	946.2
0.42482	80.9555	4302.6	0.42421	114.4194	3441.9	0.45051	165.5516	956.7
0.44963	81.2776	4311.1	0.45042	114.7779	3452.5	0.47554	165.9075	967.2
0.47512	81.6035	4322.1	0.47423	115.1017	3463.1	0.50060	166.2553	977.7
0.50018	81.9133	4333.1	0.50035	115.4478	3473.9	0.52556	166.6045	988.2
0.52559	82.2305	4342.2	0.52503	115.7641	3484.2	0.55069	166.9394	999.1
0.55019	82.5390	4352.9	0.55011	116.0774	3495.3	0.57535	167.2706	1010.4
0.57523	82.8355	4363.6	0.57496	116.3859	3505.7	0.60108	167.5986	1020.9
0.60009	83.1260	4374.2	0.60003	116.6754	3516.2	0.62559	167.8952	1031.8
0.62505	83.4019	4384.7	0.62486	116.9513	3527.2	0.65076	168.1778	1042.7
0.64992	83.6806	4395.3	0.64986	117.2250	3546.8	0.67608	168.4589	1053.6
0.67550	83.9667	4405.7	0.67510	117.4808	3556.6	0.70108	168.7282	1064.5
0.70008	84.2317	4416.2	0.72482	117.9681	3567.1	0.72570	168.9861	1075.4
0.74996	84.6807	4427.2	0.74981	118.2172	3581.8	0.75072	169.2546	1086.3
0.79846	85.2818	4446.5	0.79981	118.7772	3592.3	0.77579	169.5342	1097.2
0.85116	85.9621	4456.6	0.84853	119.4575	3603.2	0.80087	169.8538	1108.1
0.89866	86.9397	4467.1	0.89845	120.5255	3614.2	0.84974	170.5915	1118.7
0.94728	88.9547	4481.8	0.94630	122.6257	3620.1	0.89944	171.8030	1129.3
0.96922	91.1361	4492.3	0.96862	124.8987	3626.3	0.94783	174.1939	1139.9
0.98589	95.2468	4503.2	0.98565	128.9696	3632.3	0.96997	176.7669	1150.5
0.96218	92.2326	4514.2	0.96123	125.7080	3642.2	0.98686	181.5447	1161.1
0.93768	90.0310	4524.8	0.93696	123.3262	3652.1	0.96379	177.8350	1171.7
0.88620	88.1025	4535.4	0.88582	121.0879	3662.8	0.93976	174.9832	1182.3
0.83079	87.1958	4546.7	0.83026	119.9601	3673.7	0.88852	172.4131	1192.7
0.77858	86.6771	4556.6	0.77804	119.2861	3684.2	0.83261	171.1136	1203.1
0.72821	86.3208	4567.2	0.75132	119.0052	3694.8	0.78003	170.3449	1213.5
0.70165	86.1454	4577.8	0.70069	118.5739	3705.2	0.75289	170.0168	1224.2
0.67702	86.0059	4588.4	0.67626	118.3835	3815.8	0.72791	169.7578	1234.9
0.65187	85.8840	4599.2	0.64901	118.1951	3926.3	0.70249	169.5180	1245.6
0.62534	85.7643	4610.3	0.60117	117.8952	3936.9	0.67729	169.3016	1256.3
0.60130	85.6546	4620.8	0.59981	117.8558	3947.5	0.65225	169.0988	1267.6
0.60052	85.6348	4631.6	0.57311	117.7050	3958.4	0.65137	169.0556	1277.7
0.57498	85.5519	4642.4	0.52517	117.4105	3969.1	0.62558	168.8836	1288.4
0.52576	85.3618	4652.7	0.52462	117.3778	3979.7	0.57792	168.5344	1299.1
0.52514	85.3258	4663.1	0.49736	117.2174	3990.2	0.57613	168.4851	1309.8
0.51086	85.2713	4673.3	0.46245	116.9851	4000.7	0.54954	168.3018	1320.5
0.47615	85.1436	4683.6	0.44878	116.8216	4011.2	0.50284	167.9521	1331.2
0.45024	85.0188	4694.5	0.41421	116.2450	4022.3	0.50018	167.8952	1342.1
0.45009	84.9798	4705.4	0.39750	115.7751	4033.5	0.48708	167.7953	1353.2
0.44349	84.9463	4716.3	0.38684	115.5170	4044.7	0.45065	167.3166	1363.9

0.41326	84.7552	4727.2	0.38033	115.3578	4054.9	0.43665	167.1254	1374.8
0.41227	84.6781	4738.1	0.37458	115.2398	4065.1	0.40431	166.2136	1385.7
0.39885	84.5318	4749.8	0.36223	115.0060	4075.9	0.39906	166.0360	1396.6
0.37071	84.1318	4759.9	0.34997	114.7695	4086.3	0.38712	165.6807	1407.5
0.36178	83.9877	4770.4	0.34331	114.6464	4096.8	0.36288	165.0838	1418.4
0.34967	83.8099	4780.9	0.33736	114.5401	4107.1	0.35031	164.8155	1429.3
0.33727	83.6302	4791.4	0.33086	114.4318	4117.5	0.33787	164.5735	1440.2
0.32482	83.4635	4801.7	0.32494	114.3309	4127.9	0.32522	164.3441	1451.5
0.30004	83.1412	4812.1	0.30076	113.9670	4138.2	0.31288	164.1258	1461.8
0.25049	82.5447	4822.3	0.25094	113.2372	4148.6	0.30005	163.9058	1472.6
0.20038	81.9270	4832.4	0.20053	112.4151	4158.9	0.25278	162.8187	1483.4
0.15067	81.1875	4842.5	0.15051	111.4210	4169.2	0.20095	161.6028	1494.2
0.10080	80.1856	4853.1	0.10088	110.0911	4179.8	0.15071	160.3947	1505.4
0.07480	79.4646	4863.7	0.07476	109.1209	4190.3	0.10141	158.3814	1516.2
0.05048	78.5158	4875.6	0.05052	107.8569	4203.2	0.07501	156.9049	1527.6
